# ATLAS: A Traffic Load Aware Sensor MAC Design for Collaborative Body Area Sensor Networks

**DOI:** 10.3390/s111211560

**Published:** 2011-12-12

**Authors:** Md. Obaidur Rahman, Choong Seon Hong, Sungwon Lee, Young-Cheol Bang

**Affiliations:** 1 Department of Computer Engineering, Kyung Hee University, Gyeonggi-do 446-701, Korea; E-Mails: mdobaidurrahman@gmail.com (M.O.R.); drsungwon@khu.ac.kr (S.L.); 2 Department of Computer Engineering, Korea Polytechnic University, Gyeonggi-do 429-793, Korea; E-Mail: ybang@kpu.ac.kr (Y.-C.B.)

**Keywords:** collaborative sensors, Wireless Body Area Network (WBAN), traffic load, IEEE 802.15.4, superframe, energy, throughput, and delay

## Abstract

In collaborative body sensor networks, namely wireless body area networks (WBANs), each of the physical sensor applications is used to collaboratively monitor the health status of the human body. The applications of WBANs comprise diverse and dynamic traffic loads such as very low-rate periodic monitoring (*i.e.*, observation) data and high-rate traffic including event-triggered bursts. Therefore, in designing a medium access control (MAC) protocol for WBANs, energy conservation should be the primary concern during low-traffic periods, whereas a balance between satisfying high-throughput demand and efficient energy usage is necessary during high-traffic times. In this paper, we design a traffic load-aware innovative MAC solution for WBANs, called ATLAS. The design exploits the superframe structure of the IEEE 802.15.4 standard, and it adaptively uses the contention access period (CAP), contention free period (CFP) and inactive period (IP) of the superframe based on estimated traffic load, by applying a dynamic “**wh**” (**wh**enever **wh**ich is required) approach. Unlike earlier work, the proposed MAC design includes *load estimation* for network load-status awareness and a *multi-hop communication pattern* in order to prevent energy loss associated with long range transmission. Finally, ATLAS is evaluated through extensive simulations in ns-2 and the results demonstrate the effectiveness of the protocol.

## 1. Introduction

In recent years, rapid advancements in micro-electro-mechanical systems (MEMS) [1] have inspired the adoption of collaborative sensor networking for the acquisition of health related information. This type of networking is known as wireless body area networks (WBANs) or simply body sensor networks (BSNs) within the wireless research community. Low-power and inexpensive sensors with low computation and communication capacity are widely used in WBANs. The basic applications of WBANs include long-term health-care, patient monitoring in a hospital environment, collection of health-related information for workers in hostile and life-threatening areas (e.g., soldiers, deep-sea and space explorers, *etc*.) [2]. It is noticeable that the WBAN applications necessitate both low-rate periodic monitoring (*i.e.*, observation) data and high-rate traffic including event-triggered bursts. Therefore, communication protocols for WBANs should allow for dynamic operation in response to the such fluctuations and variations in application traffic.

In a typical WBAN, both implanted and body-attached physical sensors are deployed inside and outside of the human body that collaboratively monitor a user’s health condition and transmit the observed data to a powerful external gateway such as a PDA, smart phone, smart watch, *etc*., in order to facilitate timely and effective action. The collaborative WBAN model could have either single-hop star topology [3] or multi-hop tree and mesh topologies [4–8] which are rooted at the gateway. However, the medium access control (MAC) operations of the nodes in such WBAN topologies are very challenging for several reasons. First, the resource-constrained sensors have difficulty in dealing with the considerable variation in the data generation rate due to the scarcity of node energy and computation power. For example, in addition to low observation traffic, few low-rate physical sensors used for monitoring heart beat, blood pressure, glucose level, body temperature, *etc*., often generate time-critical emergency data. Furthermore, the sensor used for real-time streaming of ECG signals always generates data at a high rate. Second, some low-rate applications require energy conservation whereas the event-triggered and high-rate applications demand high-throughput, and minimal delay is expected in both cases. Hence, energy conservation should be the primary concern during low-traffic periods, whereas a balance between satisfying high-throughput demand and efficient energy usage is necessary during high-traffic times. Finally, the limited channel capacity and internal and external interferences in a dynamic wireless environment impose an unexpected and excessive burden on the execution of MAC operations. Therefore, aWBAN medium access control protocol needs to be designed in a scalable and dynamic fashion in order to simultaneously achieve *better energy-efficiency* and *high throughput* while *minimizing the delay*.

The existing efficient MAC protocols are broadly categorized in [9] as *low power listening* (LPL), *contention-based* and *time division multiple access* (TDMA). In LPL protocols [10,11], a receiver periodically wakes up from inactive (sleep) mode and checks for a potential preamble signal from the sender. If the preamble is available then receiver stays awake for data reception, otherwise it goes back to sleep. The LPL protocols are ineffective for WBANs since the preamble transmission and periodic sampling are not suitable for energy constraint sensors. However, the receiver-initiated concepts [12,13] have gained a favorable reputation because it facilitates energy conservation by minimizing the medium occupancy time of the sender’s preamble. In a contention-based protocol such as CSMA/CA, the main sources of energy inefficiency are idle listening, overhearing, collision and protocol overhead [14]. Under a low traffic environment, the low-load adaptive contention and sleep approach would work well in terms of reducing medium access latency and making the best use of channel capacity. Hence, during low traffic periods in a WBAN, adoption of such an approach instead of TDMA may yield a beneficial trade-off between energy and delay as well as efficient capacity-utilization [15]. Finally, in time-synchronized and superframe oriented TDMA protocols [3,16–18] and in the IEEE 802.15.4, time-slots are assigned using control packets (*i.e.*, beacons). Although so far TDMA is the best suited MAC strategy for WBAN, during low-rate traffic periods the overall achievable capacity utilization is drastically decreased with existing TDMA protocols. Moreover, the TDMA approach is mostly limited to perform in a WBAN with single-hop star topology, where extra energy costs are incurred for long range transmissions between the sensors and gateway. Conversely, cluster-based multi-hop communication [4,19] could serve as a potential solution to overcome the energy related shortcomings of single-hop based TDMA protocols. Note that the multi-hop communication architecture in few recent studies [6–8] justify the potential use of such communication model for WBAN.

Motivated by the observations described above, in this paper we design **A T**raffic **L**oad **A**ware **S**ensor (ATLAS) MAC protocol for collaborative body area sensor networks, more specifically for WBAN. The ATLAS protocol is a beacon-enabled, superframe oriented MAC protocol developed according to the IEEE 802.15.4 standard. This protocol efficiently and adaptively exploits the properties of contention access period (CAP), contention free period (CFP) and inactive period (IP) of the superframe of IEEE 802.15.4, while taking the traffic load-status of the network into account. The contributions of the ATLAS MAC protocol are as follows: (i) A new load adaptive complete medium access solution is introduced for WBAN in which the receiver-driven approach is used for the protocol operation and it provides better energy-efficiency, high capacity-utilization (*i.e.*, throughput) and minimal delay; (ii) An innovative load estimation and differentiation technique is proposed that uses the network capacity as the most decisive parameter to justify the congestion (load) status of the network; (iii) The network model consists of a multi-hop communication pattern that is very new in terms of WBAN MAC design; (iv) Finally, the performance of ATLAS is evaluated through extensive simulations in ns-2 [20] and compared in detail with the existing schemes.

The rest of the paper is organized as follows. Section 2 discusses relevant literature on existing WBAN MAC protocols. Section 3 introduces the problems with the exiting schemes and the motivation of this study. The preliminary design considerations are described in Sections 4 and 5 presents the detailed protocol operations of ATLAS. Section 6 describes performance evaluations by comparisons with existing schemes, and finally conclusions are offered in Section 7.

## 2. Related Work

Energy-efficient MAC development for WBAN is a very prominent field of research and is receiving increasing attention. In a WBAN survey [9] we have found that amongst the well-known protocols, IEEE 802.15.4 standard is one of the earliest MAC proposals for WBANs. In this protocol, a beacon-enable or non-beacon enable mode decides how to execute the medium access operation. In beacon-enable operations, a central node (*i.e.*, gateway) serves as the coordinator for time-synchronization and superframe management. The superframe structure consists of an initial beacon, a contention access period (CAP) for CSMA/CA, a contention free period (CFP) for TDMA and an inactive period (IP) for sleep. However, the fixed CAP and CFP of IEEE 802.15.4 limit adaptive operation with fluctuations in the traffic load. During low traffic periods the fixed CAP and CFP result in higher energy consumption and low capacity utilization, respectively. Conversely, in a recent analysis [21] it is found that the non-beacon enable mode of IEEE 802.15.4 is suitable for prolonging the life time of a network with very low traffic (*i.e.*, periodic observation data). However, in reality consideration for only low-rate data transmission in WBAN is a very limited and infeasible assumption.

A number of studies in the literature extend the concept of the IEEE 802.15.4 protocol [9]. The LDTA-MAC [3] is one such extension that enables the transmission of emergency traffic over the network. However, the protocol does not address the dynamic and diverse characteristics of the traffic load and lacks in traffic adaptability. We have expounded on the shortcomings of LDTA-MAC in Section 3. The scheme proposed in [16] is an energy efficient pure TDMA protocol that is developed mainly for handling a large amount of data. However, this protocol is implemented with a fixed topology system and suffers from low capacity utilization when low-rate traffic exists in the network. A battery-aware TDMA protocol is proposed in [18] considering the node energy discharge, dynamic wireless channel and node buffer model characteristics. Although this protocol is able to extend the network life-time, it cannot prevent congestion losses due to buffer overflow. Recently, a heart beat driven H-MAC protocol [17] is proposed for WBANs in which each sensor must be synchronized with the human heart beat rhythm. However, the synchronization and its maintenance in H-MAC are very complex. There are a few more WBAN MAC protocols such as reservation-based dynamic TDMA (DTDMA) [22] and BodyMAC [23].

Review of the literature clearly indicates the need for a traffic load-aware WBAN MAC protocol that would work energy efficiently and can meet high-throughput demand in a dynamic traffic environment. To the best of our knowledge, there is not yet such a scheme for WBAN and we believe our proposed ATLAS protocol would meet these needs.

## 3. Problem Description and Motivation

As of now, only the single-hop based star topology is considered in existing WBAN MAC protocols, such as those described in [3,17] and IEEE 802.15.4. However, the sensors in this type of topology are bounded to make long range direct transmissions to the gateway, thereby consuming more energy. As the sensors are typically deployed throughout the body, the distance between each sensor and the gateway varies significantly. It is obvious and usual that the amount of energy required for data transmission increases as the distance between each sensor and the gateway increases [4,8], since transmission energy consumption is directly proportional to the distance. In contrast, a multi-hop communication pattern can avoid such long range transmission oriented energy loss and potentially can be implemented in WBAN arena for energy efficiency. It is noteworthy that consideration of multi-hop communication is still unrevealed in designing the WBAN MAC. However, the protocols those discussed in [4,7,8] can potentially form a multi-hop clustered communication model for WBAN. Furthermore, the recent studies in [6,8] justify the multi-hop communication model for WBAN with the idea of “intra-BAN communication” comprising of *communications between body sensors* and *communications between sensor and gateway*. Motivating by these observations, in this study we are encouraged to implement the multi-hop clustered communication pattern for a WBAN MAC protocol.

**Figure 1.**
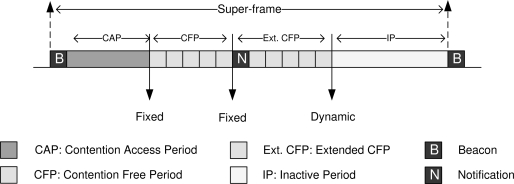
Superframe structure of LDTA-MAC.

The shortcomings of the IEEE 802.15.4 protocol are evident in the recently developed TDMA-based LDTA-MAC protocol [3]. As shown in Figure 1, the superframe structure of LDTA-MAC includes a fixed contention access period (CAP), a time-slotted fixed contention free period (CFP) along with a dynamic extended CFP and an inactive (sleep) period. From the gateway node, a *beacon* at the beginning of each superframe synchronizes the other nodes and a *notification* after the first CFP allocates the time-slots of the extended CFP (*i.e.*, upon receipt of the CFP slot request during CAP by the sensors). Although the LDTA-MAC protocol provisions for the quick transmission of emergency traffic, the given approach seems to be inefficient in several aspects, particularly in terms of adaptability to the changes in the traffic load. During the CAP in LDTA-MAC, sensors are allowed to send the data along with the CFP slot request to the gateway and maintain the inactive period. However, since the CAP is fixed for LDTA-MAC, this would lead to unfair and inappropriate allocation of the time slots of extended CFP during high traffic periods, because at such network instance some sensors might not be able to send the CFP slot request due to the heavy traffic oriented contentions. Hence, LDTA-MAC exhibits low throughput and low channel utilization when the traffic load is high. Additionally, when the traffic load is low, the fixed CAP remains under-utilized unless it is appropriately set to be a very short period prior to network setup, which is logically not feasible. Moreover, if the CAP is set to be moderately long, this could lead to wasted energy at the gateway due to unnecessary medium listening under low traffic condition. Finally, as mentioned above, the LDTA-MAC is limited to perform in the single-hop star topology, experiencing more energy cost for long range direct transmissions [4,8].

The shortcomings of LDTA-MAC highlight the fact that the superframe components of the IEEE 802.15.4 MAC protocol should be adjusted to operate adaptively according to the traffic load characteristics and network demands (e.g., energy efficiency, capacity utilization, *etc*.). These observations on existing WBAN MAC protocols have encouraged us to design the ATLAS protocol as a more generalized and diversified traffic load adaptive MAC solution for WBAN. This protocol is geared toward minimizing energy consumption during low-rate traffic and maximizing the data throughput during high-rate traffic condition in WBAN. Furthermore, the ATLAS protocol is designed to minimize the delay and maximize the channel capacity utilization.

## 4. System Model and Preliminaries

### 4.1. Network Model

**Figure 2.**
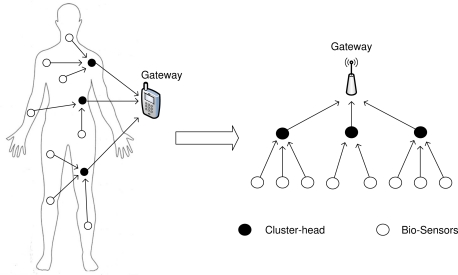
Network model for WBAN.

The proposed ATLAS-MAC assumes that several sensors are attached or implanted on/in a human body. Unlike existing TDMA MAC protocols for WBANs, we consider a clustered network as shown in Figure 2 where each sensor node belongs to a cluster and transmits the acquired data to the gateway through a respective cluster-head. Hence, the proposed MAC is designed for two types of communication [6,8], namely, *cluster-head to gateway* (Ch-to-G) and *sensor to cluster-head* (S-to-Ch).

Assume there are *N* nodes, *C* clusters and only one gateway node (denoted by *G*) in the network. The *i*-th cluster is identified by *C_i_* and its cluster-head is given by *Ch_i_*, such that *Ch_i_* ∈ *N*. The *j*-th sensor node in the *C_i_* cluster is denoted as $S_{j}^{i}$. Based on the IEEE 802.15.4 standard, the protocol operational time is divided into consecutive time intervals, alternatively called a superframe or cycle (given by *T_c_*).

### 4.2. Assumption

The data communication pattern in ATLAS is receiver-driven [12], where a beacon packet (given by *B*) is used to initiate any sort of communications. The network is assumed to be time-synchronized and the beacon includes the timing information. Moreover, several control parameter such as traffic load status, requests for an explicit CFP slot for further data traffic are implicitly piggy-backed at the packets used for protocol operation.

In ATLAS, all the devices communicate using an identical, half-duplex, low-cost single shared wireless radio. The routing process is circumvented in ATLAS, instead a static approach is used to generate a fixed route between each sensor to the gateway via one cluster-head. Hence, the routing layer tasks such as cluster formation and cluster-head selection are beyond the scope of this study, and protocols discussed in [4,19] can be adopted for this purpose. Note that the multi-hop path between sensors to the gateway is bi-directional.

## 5. The ATLAS MAC Protocol

The basic aim of the ATLAS protocol is to ensure the medium access control of the network nodes, taking the estimated traffic load status (*i.e.*, low, moderate, high and overload) into account. The details of the ATLAS protocol are described in the following subsections.

### 5.1. Traffic Load-Status Differentiation

The radio capacity and traffic load are very closely coupled in a single shared wireless medium. Several issues such as contention, collision, internal and external interferences, hidden terminal problems and so forth have a direct impact on the network’s traffic load. In ATLAS, we have estimated multi-level traffic load-status by taking the radio capacity usage into account. We are interested in estimating the traffic load at the cluster-head because, in the given network model (in Section 4.1), the cluster-head is more broadly representative of the dynamic network conditions and is a good indicator of any changes in the network.

We have included a simplified traffic load estimation technique in which each cluster-head measures the load based on its capacity usage. Furthermore, we classify its traffic load status into the following categories: *low-load*, *moderate-load*, *high-load*, and *over-load*. Due to the variations in data traffic rates, each cluster-head needs to measure the load over an appropriate time interval. To accomplish this we use the same superframe/cycle duration *T_c_* as the measurement period, and the *load index*, *L_i_*, of cluster-head *Ch_i_* is given by,

(1) $$L_{i}=\frac{T_{c}\times ({ar}_{i}^{s}+{fr}_{i}^{s}+cr_{i})}{c\times \eta \times T_{c}}=\frac{ar_{i}^{s}+{fr}_{i}^{s}+cr_{i}}{c\times \eta }$$

where *c* is the radio capacity and we use *η* as the maximum anticipated utilization of the capacity *c* due to the dynamic wireless environment in WBAN. Intuitively, *η* deduces the nominal capacity [24] of each cluster-head. Using the random network capacity analysis in [25] and our simulation parameters, the nominal capacity of each node is derived as a pre-task, where we set *η* = 0.47 (see Section 5.3). Moreover, ${ar}_{i}^{s}$ and ${fr}_{i}^{s}$ represent the successful packet’s arrival and forwarding rates, respectively, and *cr_i_* is the collision rate at *Ch_i_*. Using Equation (1) we calculate the total amount of traffic (*i.e.*, arrived, forwarded and collided) at *Ch_i_* during the *T_c_* period. Note that a cluster-head considers only the successfully received and transmitted packets per unit time (*i.e.*, seconds) to calculate the ${ar}_{i}^{s}$ and ${fr}_{i}^{s}$, respectively. In contrast, the *cr_i_* expresses the number of collisions detected by *Ch_i_* (either in sending or receiving) per-unit time; hence, the interference effects at *Ch_i_* due to neighbors, interferers and hidden terminals are reflected by the measured *cr_i_*. The load estimation does not consider the losses due to the *bit error rate* (BER) since the BER probability always remains same in an identical wireless environment.

The categorization of the load-status based on the *L_i_* is a critical task because the choice of load transition values requires a trade-off between capacity utilization and congestion/load awareness (*i.e.*, the queue length of a node). A small value of *L_i_* keeps the network in a low-congestion and contention state, in which the capacity utilization would be at a minimum. Conversely, although a large transition value can ensure better use of the capacity, it may quickly overload the network, resulting in more congestion (*i.e.*, buffer overflow) and collision losses. Therefore, we have chosen suitable load transition values such that optimal capacity utilization and congestion avoidance can be guaranteed.

Assume that the queue length of a cluster-head *Ch_i_* is *q_i_* and can hold 40 packets. We define lower and upper thresholds for *q_i_*, given by $q_{i}^{l}$ and $q_{i}^{u}$, respectively. In ATLAS, the values for $q_{i}^{l}$ and $q_{i}^{u}$ are set to be 3 and 8, respectively; and the analysis in [26,27] validates these assumptions. In our extensive simulations, we have determined that when *L_i_* exceeds 0.92, the queue length of a cluster-head always remains beyond $q_{i}^{u}$. Conversely, for a *L_i_* value less than 0.74, the queue length stays below $q_{i}^{l}$. Therefore, we select the former and latter observations for the *over-load* and *low-load* states, respectively; whereas, for the *moderate-load* and *high-load* states we evenly divide the range between 0.74 and 0.92. Furthermore, in selecting each load-status, a queue length constraint is also associated with the selected load index (*L_i_*) values.

Table 1 shows the categories of the loads along with their differentiation conditions with respect to the estimated *L_i_* for *Ch_i_* and an expected queue length constraint. The ATLAS assumes that if a cluster-head has *L_i_* ≤ 0.74 or its queue length satisfies $q_{i}\leq q_{i}^{l}$, then it is in a low-load state; whereas either for *L_i_* > 0.92 or $q_{i}\geq q_{i}^{u}$ the cluster-head is in an over-load state. The moderate-load and high-load are evenly categorized within the range between minimum and maximum bounds of *L_i_* for low-load and over-load, respectively, and also should satisfy a common expected queue length constraint, such that $q_{i}^{l}<q_{i}<q_{i}^{u}$.

Upon detecting the load, each cluster-head attaches two load-status notification (*LN*) bits at every transmitted data and beacon packet. Thus, by obtaining the *LN* of the cluster-head, the gateway and sensor nodes become aware of the overall traffic load condition of the surrounding environment and similarly share the information with other sensors in their vicinity. Consequently, the traffic load-aware and receiver-driven communications are controlled either by the gateway node or the cluster-heads.

### 5.2. Medium Access Design

The overall medium access operations of ATLAS follow a hybrid-approach based on the type of communication (*i.e.*, Ch-to-G or S-to-Ch) and load-status information (*i.e.*, low, moderate, high and overload). In Ch-to-G communication, the gateway node plays the coordinating role in selecting the mode of medium access according to the available traffic load-status information. More specifically, using the “**wh**” (whenever which is required) approach, the gateway exploits the CAP, CFP, and IP modes or intervals of a superframe. Conversely, in S-to-Ch communication, the medium access pattern is asynchronous and cluster-head controlled. In this case, the communication depends upon the mode of medium access operation between Ch-to-G.

#### 5.2.1. Cluster-Head to Gateway (Ch-to-G) Communication

**Figure 3.**
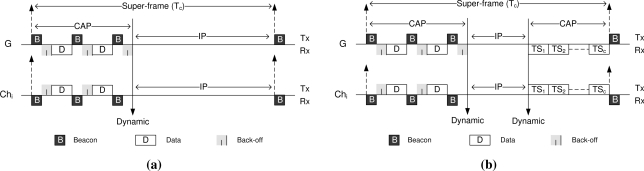

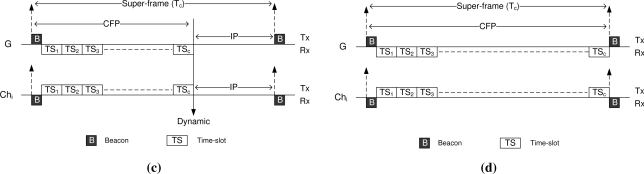
Cluster-head to gateway (Ch-to-G) communication snapshot: **(a)** Low load; **(b)** Moderate load; **(c)** High load; and **(d)** Overload.

As mentioned earlier, upon estimating the traffic load-status, each cluster-head piggy-backs the load index information (*i.e.*, *LN*-bits) at each outgoing beacon and data packet. Hence, the gateway node *G* is well aware of the traffic load of the network and coordinates the medium access at each superframe accordingly. Note that the very first initial beacon (*B*) from node *G* at each superframe serves three purposes: (i) includes clock information for time synchronization; (ii) acts as a data request message; and (iii) serves for time-slot assignment purposes. The MAC operations for Ch-to-G are described below for each traffic load state:

- *Low-load* (Use of CAP and IP): In low-load state, the gateway *G* operates only in contention access period (CAP) mode, as shown in Figure 3(a). To do so, node *G* sets the data request field at the initial transmitted beacon *B* and waits for a time-out period (given by *T_to_*) for possible data reception. The *T_to_* period includes the maximum back-off interval plus one extra time slot as a guard time. However, in reply to the beacon if any cluster-head transmits a data packet, then node *G* accepts it and acknowledges its acceptance with a data-Ack beacon (setting the ACK bit in the beacon packet). In this case, the later beacon acts as another request for data reception in CAP mode. After a data request beacon transmission, the gateway always waits for *T_to_* to receive data, otherwise it would goes into inactive period (IP) mode until the next superframe.
  In contrast, at the beginning of a superframe, each cluster-head *Ch_i_* listens to the medium for the initial beacon *B* from *G* and determines the mode of medium access operation. A cluster-head with data remains awake until it is able to send the data or goes into IP otherwise. However, if the data request bit is found set in the received beacon, each *Ch_i_* performs a random back-off to avoid collision with other potential cluster-heads (senders). A cluster-head with early back-off expiration sends a data packet and waits for a *short intra-frame space* (SIFS) to receive a data-Ack from the gateway. If any cluster-head loses the contention, it pauses the back-off and updates the *network allocation vector* (NAV) until the ongoing data transmission finishes. Another data-request beacon (*i.e.*, data-Ack for the previous transmission) from the gateway resumes the back-off for such cluster-heads. Hence, the contention access period (CAP) in ATLAS is dynamically adjusted, where the inactive period of the nodes starts whenever there is no data available to send or receive. Unlike LDTA-MAC [3], the given load-adaptive approach is able to maximize the capacity utilization under low traffic.
- *Moderate-load* (Use of CAP, IP and CFP): At moderate-load, the gateway sequentially and dynamically uses the contention access period, inactive period and contention free period in a single superframe (Figure 3(b)). Furthermore, during CAP, a cluster-head is allowed to set a request in the data packet header for a guaranteed CFP time-slot (*i.e.*, given by *TS_c_*). The rationale behind using both CAP and CFP is to ensure maximum capacity utilization. This is because, as the load increases the contention also increases, hence a few cluster-heads might require additional slots for further data transmission in addition to CAP.
  The CAP operation in this case is similar to that in the low-load state. However, the inactive period starts here following the CAP and lasts until the CFP. The CFP in this current superframe continues until the start of the next superframe. The assignment of guaranteed time-slots in ATLAS during moderate-load is very simple and straightforward. Assuming *C* time-slots are required in the CFP of moderate-load status of the network. Hence, whenever the gateway receives a time-slot request from a cluster-head, it simply assigns the *C*-th time-slot *TS_c_* using the data-Ack beacon during CAP. The *TS_c_* slot is the closest time-slot to the next superframe. When there is any additional request for a slot, the gateway assigns the *TS*_*c*−1_ slot and so on. As a result, the CAP and CFP move toward the middle of the superframe, and the rest of the period is maintained as the IP. Thus, all the periods or components of the superframe are dynamically adjusted in moderate-load status and can maintain a fair contention environment.
- *High-load* (Use of CFP and IP): Upon detecting high-load traffic in the network, the gateway switches to the CFP mode. Hence, in the initial beacon (*B*) it fairly assigns a guaranteed time-slot to each of the cluster-heads (Figure 3(c)). Each cluster-head becomes active only in the assigned slot for data transmission and remains inactive otherwise. The transmitted data at each time-slot is acknowledged by the gateway. Thus, if any cluster-head requires multiple time-slots for transmission, this request can be made through the data packet header and an additional time-slot can be allocated for the same cluster-head using the data-Ack beacon of the current slot.
  Following all slot assignments and data receptions, it is expected that the rest of the superframe would be the IP. Therefore, the CFP and IP are also dynamically adjusted under high-load state.
- *Over-load* (Use of CFP): When the network load reaches the over-load state, it is anticipated that the network capacity reaches its saturation level. Hence, in order to achieve maximum throughput the gateway uses the full CFP mode throughout the superframe (Figure 3(d)). The IP is useless in such a state, since it might cause packet dropping due to buffer overflow at the cluster-heads. The ATLAS protocol stops packet reception from the sensors at the cluster-head, unless the load-status drops to high-load status or below (see S-to-Ch communication in the next section). This concept is similar to the rate-control techniques proposed in [24,28] where the source nodes throttle down the rate while sending their data to the heavily loaded node. In assigning the time-slots in CFP, the gateway maintains weighted fairness according to the estimated load status of the cluster-heads. More specifically, the cluster-head that have a higher load would be assigned more time-slots than the relatively lower loaded cluster-heads.

#### 5.2.2. Sensor to Cluster-Head (S-to-Ch) Communication

The medium access control for the S-to-Ch communication is somewhat dependent on the traffic load-status and the mode of MAC operation currently used in Ch-to-G communication. However, the operational procedure is asynchronous and also receiver-driven. Although the sensor nodes are synchronized and maintain the same superframe structure, they operate asynchronously during the available CAP and IP modes of the superframe. Similar to the basic receiver-initiated MAC operation [12], whenever a sensor node has some data to send, it maintains *low power listening* (LPL) and waits for the receiver’s beacon (*i.e.*, the data request message from the cluster-head). In contrast, performing a random back-off, the cluster-head sends the data request beacon to the sensors only during the available CAP and IP of the superframe. Thus, the S-to-Ch data transmission would not interrupt the CFP operation of the superframe during Ch-to-G communication. Note that if the medium is found to be BUSY while attempting beacon transmission, the cluster-head waits until *T_to_* plus a data packet transmission time and makes another attempt for beacon transmission. In ATLAS, the maximum bound for such a beacon transmission attempt is set to four per superframe, considering the short packet retry limit of the CSMA/CA protocol.

The basic MAC operations for S-to-Ch communication is cluster-head controlled and described using the pseudo code of Algorithm 1. As given in lines 1–13, under low (*LN* = 00) and moderate (*LN* = 01) loads when the Ch-to-G communication follows either CAP or IP mode, cluster-heads contend for data-request beacon transmission and accordingly acknowledge the data reception from the sensors. If there is no data, a cluster-head simply sleeps or performs LPL for possible beacon reception from the gateway. A similar procedure is followed by the cluster-heads when the network traffic load is high (*LN* = 10) (lines 14–26). The only exception is in line 22, the cluster-head simply goes to sleep when there is no data to send. Finally, when the load-status is over-load (*LN* = 11) (lines 27–29), the cluster-heads defer the data request beacon transmission to the sensors. This approach prevents data reception at the cluster-heads, thereby avoiding buffer drops; this is a type of trade-off that avoids packet drops due to buffer overflow at the cluster-heads.

**Algorithm 1:** Medium Access Control (at each cluster-head *Ch_i_* ∈ *N*).

1.	**while** (LN == 00 || LN ==01) && (MAC Mode == CAP || MAC Mode == IP) **do**
2.	**if** (Medium == IDLE && Back-off == FINISH) **then**
3.	**Send**(Beacon Tx); /* Data Request = 1, ACK = 0 */
4.	**while Wait**(*T_to_*) **do**
5.	**if** (Packet Received) **then**
6.	**Send**(Beacon Tx); /* Data Request = 1, ACK = 1 */
7.	**goto** line 4;
8.	**else**
9.	Sleep = TRUE || LPL = TRUE
10.	**end if**
11.	**end while**
12.	**end if**
13.	**end while**
14.	**while** (LN == 10) && (MAC Mode == IP) **do**
15.	**if** (Medium == IDLE && Back-off == FINISH) **then**
16.	**Send**(Beacon Tx); /* Data Request = 1, ACK = 0 */
17.	**while Wait**(*T_to_*) **do**
18.	**if** (Packet Received) **then**
19.	**Send**(Beacon Tx); /* Data Request = 1, ACK = 1 */
20.	**goto** line 17;
21.	**else**
22.	Sleep = TRUE
23.	**end if**
24.	**end while**
25.	**end if**
26.	**end while**
27.	**while** (LN == 11) **do**
28.	**Defer**(Beacon Tx);
29.	**end while**

The overall MAC operations of ATLAS for the Ch-to-G and S-to-Ch communications are totally dynamic and solely depend on the estimated traffic load. This load adaptive feature of ATLAS helps to achieve the primary goals of energy efficiency and high-throughput in diverse traffic environments.

### 5.3. Parameter Selection

#### 5.3.1. The Value of *η*

The *η* is used to deduce the nominal portion of the radio capacity *c* for each cluster-head (Section 5.1), which we derived from the given per-node capacity (denoted by *λ*) of a random network, as deduced in [25],

(2) $$\lambda <\frac{k\times R_{\mathrm{tx}}}{\rho }\times \frac{1}{L}=\frac{c\times R_{\mathrm{tx}}}{n\times L}$$

where, *k* is a constant, *ρ* is the node density, *n* is the number of nodes within the transmission range $(R_{\mathrm{tx}})\;n=\rho \pi R_{\mathrm{tx}}^{2}=\frac{20}{30\times 30}\times \frac{22}{7}\times {\left(15\right)}^{2}\approx 15$), and *L* is the expected physical path length in the network. The value of *ρ* depends on the area of the network given by *A*, and *A* = 30×30 square meters here. We also use a few of our simulation parameters to deduce an upper bound for *η* using Equation (2). Thus, for a deployment of 20 nodes in *A* with *c* = 250 kbps, *R_tx_* = 15 m, *n* = 15, and *L* = 2, we obtain an upper bound of *η* that is equal to 0.47.

## 6. Performance Evaluations

### 6.1. Simulation Setup and Performance Metrics

The performance of ATLAS is evaluated through extensive simulations using the ns-2 [20] simulation tool. A network of 30 × 30 *m*^2^ area is used, where 20 sensor nodes are deployed in a uniform random distribution. A gateway-rooted 2-hop routing tree is pre-established using the deployed nodes where the first-level nodes serve as the cluster-heads. The transmission and interference ranges of the nodes are set at 15 m and 33 m, respectively. In WBAN, the sensors are mostly located on the human body and we have chosen a large transmission range and network size to simulate the increasing demands for the both in-body and on-body nodes deployment and network scalability. Such design choice can be validated by the CodeBlue prototyped medical sensor network platform [29]. Furthermore, the radio capacity is set at 250 kbps. Table 2 summarizes the simulation parameters, most of which are extracted from the implementation of LDTA-MAC [3] in conjunction with the IEEE 802.15.4 standard. Finally, to yield more stable results, an average of fifty simulation executions is performed, each of that is 100 s long.

In this study, we compare the performance of ATLAS with LDTA-MAC and IEEE 802.15.4, since both of these protocols include the superframe oriented CAP, CFP and IP modes. Both the LDTA-MAC and IEEE 802.15.4 protocols are implemented for a single-hop star topology and sensor nodes make direct transmissions to the gateway. In ATLAS, the MAC operation modes are conveyed through the initial beacon, including the time-slot assignment of the current superframe. Moreover, at each data-Ack beacon we make provisions for assigning time slots and announcing other network parameters. Note that for LDTA-MAC, in addition to the beacon frame, an extra notification frame is used for time-slot assignment.

To analyze the performance of the protocols, we have used the following metrics: (i) *Average duty cycle*—the ratio of the per cycle average active time to the entire cycle time, expressed as a percentage; (ii) *Energy consumption*—the average energy consumption of all the nodes of the network, expressed in Joules; (iii) *Aggregate throughput*—the sum of the sizes of the total packets received by the gateway per unit simulation time; (iv) *Delivery ratio*—the ratio of the total number of packets received by the gateway to the number of packets generated at the sensor nodes; and (v) *End-to-end delay*—the average time interval between a packet generated at a sensor and received at the gateway. Note that both the aggregate throughput and packet delivery ratio reflect the data fidelity effectiveness of the protocols. Here, the data fidelity is defined as how well the demands (*i.e.*, aggregate throughput and delivery ratio) of application data are fulfilled by the MAC protocols. The data fidelity may be hampered if significant packet drops occur in the network, thereby resulting in low aggregate throughput and delivery ratio.

### 6.2. Performance with Varying Traffic Loads

**Figure 4.**
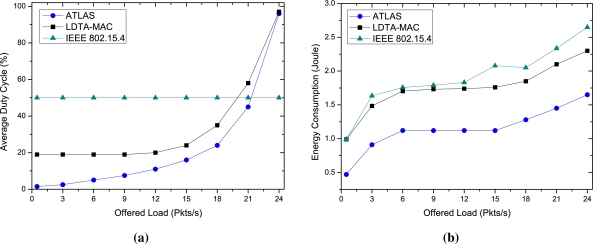
Energy efficiency over diverse traffic loads: **(a)** Average duty cycle; and **(b)** Average energy consumption.

In the performance evaluation, we first simulate diverse traffic rates from all the deployed sensor nodes. In Figure 4, the energy efficiency of ATLAS is compared with the other protocols as a function of traffic load. As delineated in Figure 4(a), the duty-cycle of the protocols increases with increased data generation rate. At a low load, the duty-cycle in ATLAS rises marginally; however, as the load increases, the sensor nodes operate more in active mode in order to handle the data due to the traffic load adaptability. In contrast, the IEEE 802.15.4 protocol maintains a fixed duty-cycle of 50%, irrespective of the traffic load. In LDTA-MAC, although the duty-cycle at high traffic is almost equal to the ATLAS, under low traffic it is relatively high. In terms of energy consumption, a low duty cycle is anticipated at very low traffic for energy conservation, and at a moderate traffic of 12 Pkts/s, ATLAS has only an 11% duty-cycle. Conversely, LDTA-MAC has a 20% duty-cycle under the same traffic load.

The average energy consumption of the protocols is shown in Figure 4(b). According to the results, the energy expenditure for ATLAS is significantly lower than that for the other two protocols. The main reason for this is the maintenance of an adaptively long inactive period during low traffic periods as well as better energy utilization under high traffic loads. Note that as the traffic load increases the energy consumption also increases for ATLAS. The LDTA-MAC and IEEE 802.15.4 have almost identical high energy cost under low traffic conditions since they employ a fixed CAP concept, thereby increasing the energy consumption. Furthermore, the long range transmissions of the sensor nodes (in single-hop) increase the energy costs for both LDTA-MAC and IEEE 802.15.4. However, at high rates of data generation, the LDTA-MAC wastes less energy for collision drops than that of IEEE 802.15.4 due to its traffic adaptive CFP use. Still it consumes more energy than ATLAS due to the use of a fixed CAP and single-hop oriented direct communications. On the other hand, only CFP property of the superframe is exploited in ATLAS under high traffic conditions, thereby maximizing energy conservation.

**Figure 5.**
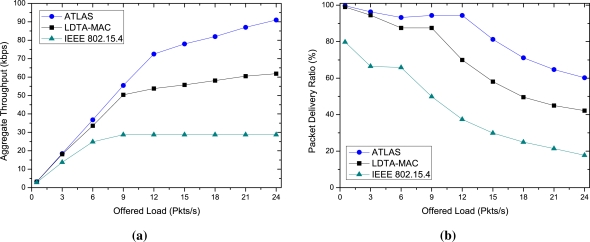
Data fidelity over diverse traffic loads: **(a)** Aggregate throughput; and **(b)** Packet delivery ratio.

In Figure 5, the data fidelity in terms of aggregate throughput and delivery ratio of the protocols are observed. As shown in Figure 5(a), even though the aggregate throughput of all the protocols increases with the traffic load, the variation is noticeable especially under high traffic loads. The proposed ATLAS protocol achieves a maximum throughput of 78 kbps at a high rate of 15 Pkts/s, and the average per node throughput is 12.18 Pkts/s. In contrast, for the same data generation rate, adaptive CFP allocation seems advantageous to LDTA-MAC as it achieves a throughput of 55 kbps with a per node throughput of 8.7 Pkts/s, which is higher than the static CAP, CFP and IP based IEEE 802.15.4 protocol, but not better than our protocol. The load specific and more appropriate use of CAP, CFP and IP operations help ATLAS to achieve a superior throughput performance compared to the other protocols.

The delivery ratio shown in Figure 5(b) also reflects the throughput performance and proves the data fidelity effectiveness of ATLAS over the other protocols. At different traffic loads, the maximum and minimum delivery ratio of ATLAS are 99.6% and 60.2%, respectively; for LDTA-MAC which are 99% and 42.18%, respectively. The delivery ratio of IEEE 802.15.4 is the worst amongst the simulated protocols since it can hardly handle the heavy traffic of the network.

**Figure 6.**
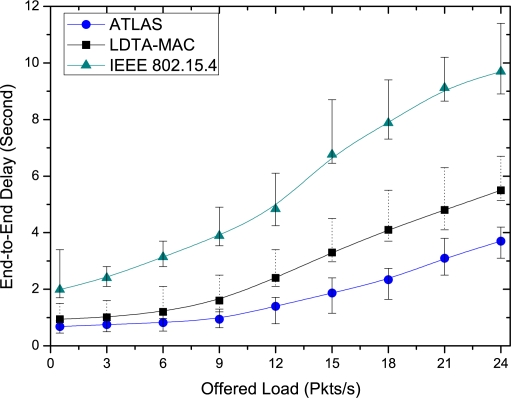
End-to-end delay over diverse traffic loads.

The proposed ATLAS protocol outperforms both the LDTA-MAC and IEEE 802.15.4 protocols in achieving lower delay. As shown in Figure 6, the end-to-end delay of the IEEE 802.15.4 is significantly higher than that in ATLAS and LDTA-MAC, since the protocol is unable to make a timely delivery of the data packets due to the high contention and collision losses at both low and high traffic. In contrast, both ATLAS and LDTA-MAC experience a lower delay under low traffic until 9 Pkts/s. However, as the traffic load increases beyond this, the delay performance of LDTA-MAC suffers due to the lack of traffic load awareness and adaptation. Conversely, the ATLAS protocol can adaptively make efficient use of the different modes of MAC operation and is thereby able to transmit the data with minimum delay, even at high traffic loads. In the graph of Figure 6, the vertical line shows the maximum and minimum delay variation of the different simulations runs, and it is evident that the ATLAS protocol has the least variation.

### 6.3. Performance with Different Number of Nodes

We vary the number of nodes in the network from 2 to 20. In this part of the simulation, each node is considered to generate data at a very high rate of 15 Pkts/s and the other simulation parameters remain the same.

**Figure 7.**
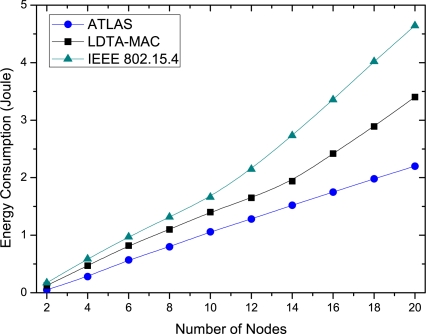
Average energy consumption over different number of nodes.

As delineated in Figure 7, the overall average energy consumption of the protocols increases proportionally with the number of network nodes. As shown in Figure 7, for various traffic loads from different numbers of deployed nodes, the proposed ATLAS has the lowest energy consumption in comparison to LDTA-MAC and IEEE 802.15.4 protocols. This is due to the efficient use of energy in handling data traffic under different load scenarios. Conversely, the LDTA-MAC and IEEE 802.15.4 are unable to adapt their operations based on the traffic load, particularly when there are many network nodes generating more traffic. Furthermore, their protocol operation includes long range single-hop transmissions, causing much more energy consumption. However, overall LDTA-MAC consumes less energy than the IEEE 802.15.4 does.

**Figure 8.**
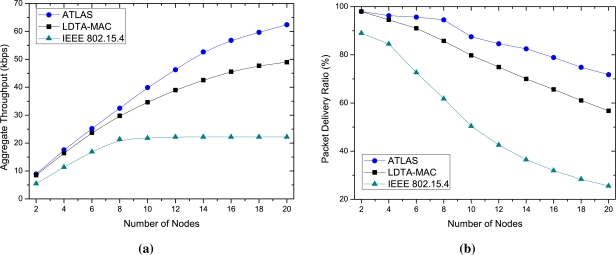
Data fidelity over different number of nodes: **(a)** Aggregate throughput; and **(b)** Packet delivery ratio.

Figure 8 illustrates the data fidelity (throughput and delivery ratio) of the protocols for different numbers of deployed nodes. As portrayed in Figure 8(a), in comparison to the other protocols, ATLAS maintains a better throughput for both lower and higher number of sources. When there are 20 nodes in the network, ATLAS ensures a successful delivery of about 70% of the packets per second. However, the percentage of successful delivery is approximately 53% and 24% for LDTA-MAC and IEEE 802.15.4, respectively. Similarly, the delivery ratio shown in Figure 8(b) indicates better results using the proposed MAC protocol. The ATLAS protocol achieves a maximum ratio of 87.5% for 10 nodes and about 71% for 20 nodes. In contrast, due to the lack of traffic adaptability in using the CAP, CFP and IP modes of the superframe, the delivery ratio of LDTA-MAC and IEEE 802.15.4 suffer considerably, especially when the network load increases due to heavy traffic from a higher number of nodes.

**Figure 9.**
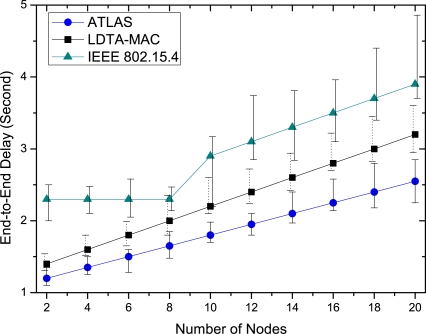
End-to-end delay over different number of nodes.

The average end-to-end delay is also observed for various numbers of deployed nodes. As depicted in Figure 9, ATLAS has a lower delay in comparison to both LDTA-MAC and IEEE 802.15.4, and the maximum and minimum delay variation is also minimal for ATLAS. However, the average delay increases linearly with the number of nodes. The interpretation of the delay result as shown in the graph is the same as that given for Figure 6 and is not repeated here. Overall, these results support the delay effectiveness of ATLAS and its suitability for MAC operations in diverse WBAN applications.

## 7. Discussion and Concluding Remarks

The superior performance of ATLAS actually comes at the cost of some protocol overhead, which are also seen in previous WBAN MAC protocols such as LDTA-MAC [3] and IEEE 802.15.4. First, the MAC operation in ATLAS depends solely on the time synchronization of the sensor in order to maintain simultaneous superframes. Thus, it requires regular timing information, which is actually piggy-backed at the initial beacon of the gateway. Second, ATLAS uses the data-Ack beacon to announce the mode of operation based on the traffic load of the network in each superframe. Finally, the proposed scheme has a few limitations in terms of its implementation in WBAN. The proposed protocol is described considering only a single-path and single gateway-rooted network topology, even though aWBAN might consist of multi-path, multi-gateway, and multi-tree topology. Moreover, ATLAS lacks in making a compatibility study with any clustering algorithm in terms of energy, which seems to be very challenging for existing energy-efficient sensor MAC protocols as well. However, we leave the implementation of these multi-concepts and the clustering compatibility study for future investigations.

The traffic load-aware ATLAS MAC protocol for body area sensor networks uses the advantageous properties of contention access period (CAP), contention free period (CFP) and inactive period (IP) of a superframe according to the IEEE 802.15.4 standard. The simulation results demonstrate that the proposed scheme performs better than the existing WBAN MAC protocols in terms of average duty-cycle, energy consumption, throughput, delivery ratio, and delay. Therefore, ATLAS is suitable for use in dynamic and diverse traffic load-oriented WBANs.

## Figures and Tables

**Table 1.** Load-status settings.

**Load Category**	**Load Differentiation Conditions**	* LN Bits
Low-load	$\begin{array}{lll} \left(L_{i}\leq 0.74\right) & \| & \left(q_{i}\leq q_{i}^{l}\right) \end{array}$	00
Moderate-load	$\begin{array}{lll} \left(0.74<L_{i}\leq 0.83\right) & \&\& & \left(q_{i}^{l}<q_{i}<q_{i}^{u}\right) \end{array}$	01
High-load	$\begin{array}{lll} \left(0.83<L_{i}\leq 0.92\right) & \&\& & \left(q_{i}^{l}<q_{i}<q_{i}^{u}\right) \end{array}$	10
Over-load	$\begin{array}{lll} \left(L_{i}>0.92\right) & \| & \left(q_{i}\geq q_{i}^{u}\right) \end{array}$	11

* Load-status Notification.

**Table 2.** Simulation Parameters.

**Parameter**	**Value**	**Parameter**	**Value**
Channel data rate	250 kbps	Buffer size	40
Payload size	32 Bytes	Super-frame period	1 s
Beacon size	11 Bytes	SIFS	192 *μ*s
PHY header	6 Bytes	CCA check delay	128 *μ*s
MAC header	8 Bytes	Time-slot length in CFP	1,920 Symbols
Back-off window	16	Symbol time	16 *μ*s
Retry limit	4	Simulation time	100 s
